# Macrophage Migration Inhibitory Factor Is Enhanced in Acute Coronary Syndromes and Is Associated with the Inflammatory Response

**DOI:** 10.1371/journal.pone.0038376

**Published:** 2012-06-05

**Authors:** Iris I. Müller, Karin A. L. Müller, Heiko Schönleber, Athanasios Karathanos, Martina Schneider, Rezo Jorbenadze, Boris Bigalke, Meinrad Gawaz, Tobias Geisler

**Affiliations:** Kardiologie und Kreislauferkrankungen, Medizinische Klinik III, Eberhard Karls Universität, Tübingen, Germany; Sapienza University of Rome, Italy

## Abstract

**Background:**

Chronic inflammation promotes atherosclerosis in cardiovascular disease and is a major prognostic factor for patients undergoing percutaneous coronary intervention (PCI). Macrophage migration inhibitory factor (MIF) is involved in the progress of atherosclerosis and plaque destabilization and plays a pivotal role in the development of acute coronary syndromes (ACS). Little is known to date about the clinical impact of MIF in patients with symptomatic coronary artery disease (CAD).

**Methods and Results:**

In a pilot study, 286 patients with symptomatic CAD (n = 119 ACS, n = 167 stable CAD) undergoing PCI were consecutively evaluated. 25 healthy volunteers served as control. Expression of MIF was consecutively measured in patients at the time of PCI. Baseline levels of interleukin 6 (IL-6), “regulated upon activation, normal T-cell expressed, and secreted” (RANTES) and monocyte chemoattractant protein-1 (MCP-1) were measured by Bio-Plex Cytokine assay. C-reactive protein (CRP) was determined by Immunoassay. Patients with ACS showed higher plasma levels of MIF compared to patients with stable CAD and control subjects (median 2.85 ng/mL, interquartile range (IQR) 3.52 versus median 1.22 ng/mL, IQR 2.99, versus median 0.1, IQR 0.09, p<0.001). Increased MIF levels were associated with CRP and IL-6 levels and correlated with troponin I (TnI) release (spearman rank coefficient: 0.31, p<0.001). Patients with ACS due to plaque rupture showed significantly higher plasma levels of MIF than patients with flow limiting stenotic lesions (p = 0.002).

**Conclusion:**

To our knowledge this is the first study, demonstrating enhanced expression of MIF in ACS. It is associated with established inflammatory markers, correlates with the extent of cardiac necrosis marker release after PCI and is significantly increased in ACS patients with “culprit” lesions. Further attempts should be undertaken to characterize the role of MIF for risk assessment in the setting of ACS.

## Introduction

Atherosclerosis is driven by sustained inflammation leading to chronic alterations of inflammatory mediators, like CRP, pro-inflammatory cytokines, metalloproteinases (MMPs), adhesion molecules and selectins [1], [2]. The interaction of endothelial cells, monocytes, macrophages and platelets plays a crucial role in upholding a pro-inflammatory and prothrombotic milieu leading to plaque instability and subsequent atherothrombotic events [3]. Additionally, there have been *in-vitro* data supporting the linkage of platelet activation, hyperaggregability and up-regulation of cytokines and chemokines involved in recruitment of inflammatory cells [4]. ACS are triggered by inflammatory response and plaque degradation as indicated by increased inflammatory processes at site of intimal rupture and increased circulating levels of MMPs during the event [5], [6]. MIF is an integral component of the host antimicrobial alarm system and stress response that promotes the pro-inflammatory functions of immune cells. A growing body of literature suggests that MIF is involved in pathomechanisms of sepsis, inflammatory and autoimmune diseases and atherosclerosis [7]–[9]. Additionally, MIF has previously been reported to be associated with signs of plaquae instability [10]. Hence, MIF might be a potential biomarker to predict the severity and the risk of ACS and to suggest therapies that target MIF pathways as attractive treatment options for cardiovascular diseases. To date little is known about the clinical impact of MIF in CAD. Hence, the aim of the present study was to further evaluate the association of MIF in a consecutive population of patients with CAD and its association with established inflammatory markers, angiographic findings and its shortterm prognostic impact.

## Materials and Methods

### Study Population and Data Acquisition

A consecutive pilot study was performed in 286 unselected patients recruited at the University Hospital Tübingen to evaluate the association of MIF, inflammatory markers and platelet function under antiplatelet therapy. All patients underwent coronary stent implantation (bare-metal and drug-eluting stents) due to symptomatic CAD. Platelet function analysis was performed in all subjects before and after receiving a 600-mg clopidogrel loading dose in addition to aspirin (ASA) pre-treatment. The study protocol was approved by the local ethical committee. Inclusion criteria comprised written informed consent and an age >18 years. A standard maintenance dose of 75 mg/day Clopidogrel + ASA 100 mg/day was prescribed for all patients in the further course. ACS was diagnosed in the presence of one of the following criteria: unstable angina (clinical symptoms and new ECG changes, but no markers of myocardial necrosis), acute myocardial infarction with markers of myocardial necrosis (TnI or creatine kinase (CK)) including ST-elevation myocardial infarction (STEMI) and non–ST-elevation myocardial infarction (NSTEMI).

### Biochemical Measurements

Peripheral venous blood samples were obtained in EDTA collection tubes at the time of PCI, centrifuged at room temperature (1500×g) for 15 min, and stored as EDTA-plasma at −80°C, until MIF levels and inflammatory markers were analysed. To assess MIF concentrations in the obtained plasma samples we used anti-human MIF ELISA, R&D Systems, Inc., Minneapolis, USA. The assay was performed according to the instruction booklet provided by R&D systems, Inc.

IL-6, MCP-1 and RANTES were determined using the Bio-Plex^©^ cytokine assay (Bio-Rad Laboratories Inc., Hercules, CA, USA) according to manufacturer’s instructions. Samples were tested for CRP using an immunoturbidimetric assay (ADVIA 1800 chemistry analyzer, Siemens Medical Solutions, Germany).

### Angiographic Analysis

Coronary angiography was performed in all patients. All angiograms were analyzed by two independent blinded investigators. Plaque rupture was angiographically defined by thrombus containing “culprit” lesion without significant angiographic stenosis.

### Statistical Analysis

A chi-square test was performed to evaluate the distribution of categorical data and a Fisher’s exact test for dichotomous analysis. A Shapiro-Wilk test was used to assess for normality of continuous variables. Normally distributed continuous data are expressed as mean ± standard deviation. For these variables means between two categories were compared with a two-tailed unpaired t-test. Non-normally distributed continuous variables are presented as median and interquartile range. Differences of non-parametric variables among categories were tested by using a Mann-Whitney-U-Test. An univariate analysis of covariance (ANCOVA) was used to estimate the effects of different factors on MIF expression. Main effects of relevant demographic factors and factors with influence on MIF expression in univariate analysis were entered into the model. All analyses were two-sided and a p-value <0.05 was considered statistically significant. Statistical analysis was performed with SPSS, Version 15 for Windows (SPSS, Inc., Chicago, IL).

## Results

### Study Cohort and Association of MIF-levels with Demographic Factors

Baseline demographics of the whole study cohort are shown and stratified according to levels of MIF under and beyond the median in table 1. Patients with high MIF levels were in general older, had more often arterial hypertension, hyperlipidemia, were more often treated with ASA, clopidogrel, statins and AT1-inhibitors and had a more preserved left-ventricular function compared to patients with low MIF-levels (≤1.96 ng/mL). Moreover, patients with high MIF levels showed significantly higher numbers of monocytes in the peripheral blood (p = 0.047).

**Table 1:** Baseline characteristics of the studied patients stratified according to median of MIF levels.

Characteristics	Total,	Low to moderate MIF (≤1.96 ng/mL)	High MIF(>1.96 ng/mL)	p-value
	n = 286	n = 143	n = 143	
Age *	67.9±13.0	65.3±14.0	70.4±11.5	**<0.001**
Female gender	84 (29.4)	38 (26.8)	46 (32.4)	0.298
Glomerular Filtration Rate (MDRD, mL/min/1.73 m^2^)*	35.2±17.4	31.2±14.0	39.0±19.5	**<0.001**
Leucocyte count (10^3^/µl)	8.7±3.1	8.4±3.2	8.8±3.1	0.136
Monocyte count (% of leucocytes)	5.0±0.2	4.5±3.6	5.3±3.1	**0.047**
**Cardiovascular risk factors – no (%)**				
Arterial Hypertension	221 (77.3)	94 (66.2)	125 (88.0)	**<0.001**
Diabetes	88 (30.8)	36 (25.4)	50 (35.2)	0.071
Hyperlipidemia	168 (58.7)	71 (50.0)	95 (66.9)	**0.004**
Tobacco use	105 (36.7)	48 (33.8)	57 (40.1)	0.269
Acute coronary syndrome	119 (41.9)	44 (31.0)	75 (52.8)	**<0.001**
LV-Function				
Slightly reduced (EF<55%)	70 (24.5)	24 (16.9)	45 (31.7)	**0.002**
Moderately reduced (EF 35–45%)	55 (19.2)	31 (21.8)	24 (16.9)	
Severely reduced (EF<35%)	49 (17.1)	34 (23.9)	15 (10.6)	
**Comedication – no (%)**				
ASA	189 (66.1)	84 (59.2)	103 (72.5)	**0.017**
Clopidogrel	87 (30.4)	35 (24.6)	51 (64.1)	**0.039**
Phenprocoumon	20 (7.0)	9 (6.3)	11 (7.7)	0.643
ACE-Inhibitors	177 (61.9)	93 (65.5)	83 (58.8)	0.222
AT1-Blockers	48 (16.8)	35 (24.6)	13 (9.2)	**<0.001**
Beta-Blockers	214 (74.8)	106 (74.6)	107 (75.4)	0.891
Diuretics	153 (53.5)	80 (56.3)	71 (50.0)	0.285
Statins	140 (49.0)	59 (41.5)	80 (56.3)	**0.013**

* mean value ± standard deviation.

† Hyperlipidemia was defined as triglycerides ≥175 mg/dl and/or LDL-cholesterol≥100 mg/dl and/or taking any of lipid lowering drugs.

ASA: aspirin.

**Figure 1:**
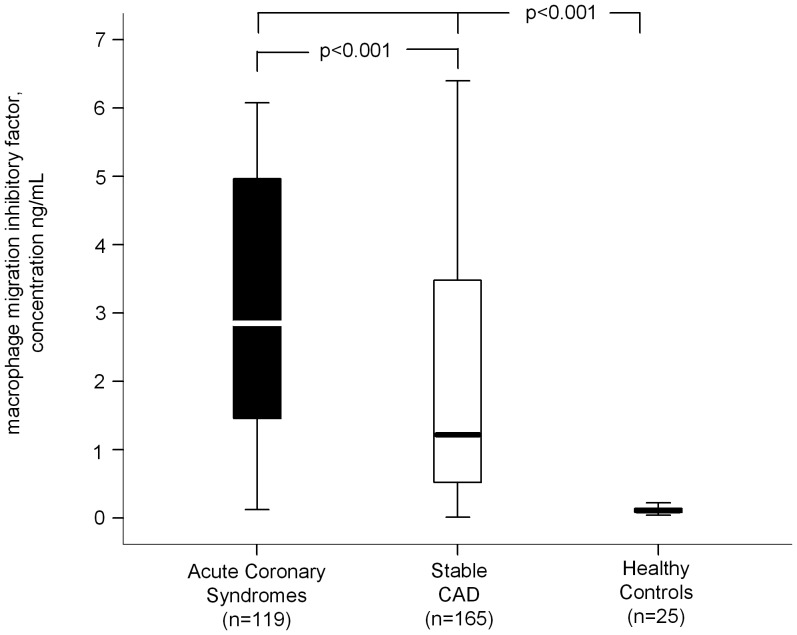
Boxplots showing MIF-levels according to acuity of CAD compared with healthy volunteers.

Patients with ACS had significant higher levels of MIF compared to patients with stable CAD and healthy volunteers (median 2.85 ng/ml, IQR 3.52 versus median 1.22 ng/ml, IQR 2.99, versus median 0.1, IQR 0.09, p<0.001, figure 1).

### Univariate Analysis of Covariance for MIF-levels and Possible Confounders for ACS

Baseline MIF-levels were independently associated with ACS in unvariate analysis of covariance (F-value 11.96, p<0.001) after inclusion of relevant variables in univariate analysis. Additionally, renal function correlated with MIF plasma levels, decreased glomerular filtration rate (F-value 23.35, p = 0.004) was independently associated with low MIF-levels. Arterial hypertension on the other hand was independently associated with higher MIF-levels (table 2).

**Table 2:** Results of univariate analysis of covariance (ANCOVA) for MIF and possible confounders in symptomatic CAD.

Coefficients		mean	95% Confidence Interval		F	Mean	Square Sig
			Lower Bound	Upper Bound			
**Age**					0.925	2.591	0.337
**Gender**	**Male**	2.29	1.87	2.70	1.49	4.18	0.223
	**female**	2.59	2.06	3.12			
**Arterial Hypertension**	**No**	2.16	1.58	2.75	3.98	11.21	**0.047**
	**Yes**	2.71	2.31	3.11			
**Acute coronary syndrome**		2.81	2.31	3.31	11.21	31.59	**<0.001**
**Stable CAD**		2.06	1.64	2.48			
**Diabetes**	**No**	2.47	2.07	2.88	0.13	0.37	0.72
	**Yes**	2.40	1.87	2.93			
**Hyperlipidemia:**	**No**	2.21	1.73	2.69	3.77	10.62	0.05
	**Yes**	2.67	2.20	3.13			
**Renal function** (MDRD, mL/min/1.73 m^2^)					6.55	18.45	**0.01**
**Reduced LV- function**	**No**	2.36	1.86	2.87	1.95	0.70	0.41
	**Yes**	2.67	2.24	3.11			
**AT1-Blockers**	**No**	2.41	2.06	2.76	0.03	0.07	0.87
	**Yes**	2.46	1.83	3.10			
**Statins**	**No**	2.35	1.87	2.83	0.51	1.43	0.48
	**Yes**	2.52	2.04	3.01			
**ASA**	**No**	2.46	1.92	2.99	0.026	0.07	0.87
	**Yes**	2.41	1.98	2.85			
**Clopidogrel**	**No**	2.39	1.99	2.80	0.04	0.11	0.84
	**Yes**	2.48	1.93	3.04			

† Hyperlipidemia was defined as triglycerides ≥175 mg/dl and/or LDL-cholesterol≥100 mg/dl and/or taking any of lipid lowering drugs.

ASA: aspirin.

### Correlation of MIF with Inflammatory Markers and TnI Release

**Figure 2:**
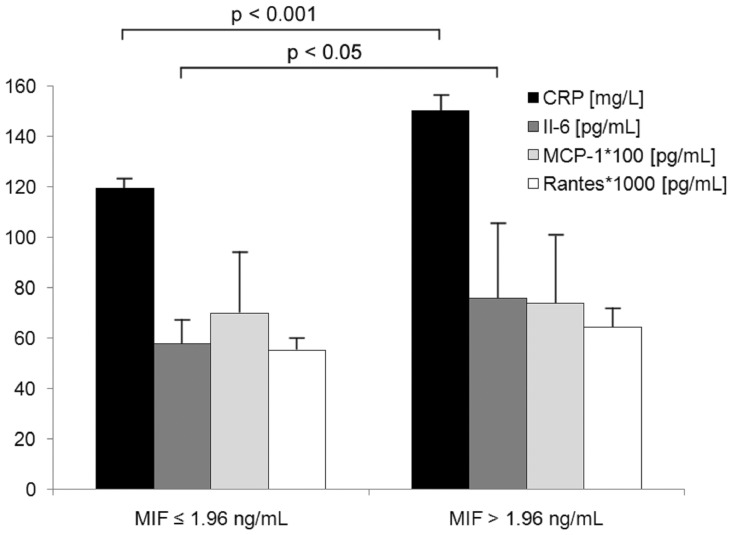
Association of inflammatory markers IL-6, RANTES, MCP-1 and CRP with MIF levels in the study cohort.

Patients with a higher amount of MIF showed significantly increased levels of CRP and IL-6, and trendwise increased levels of RANTES and MCP-1 (figure 2).

**Figure 3:**
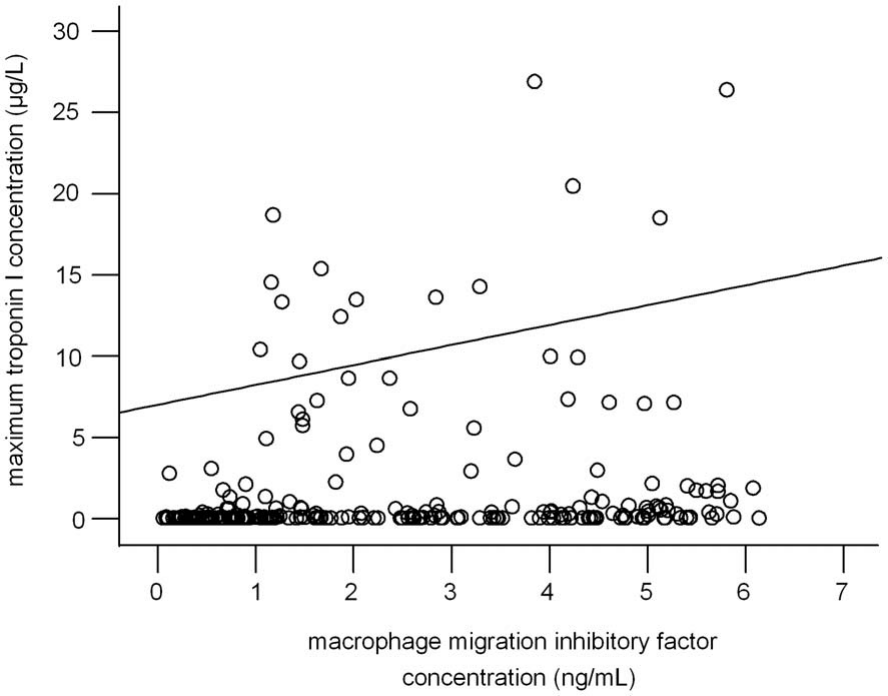
Correlation of the extent of maximum TnI release after PCI in the study cohort.

The extent of maximum TnI release after PCI moderately correlated with MIF-expression in the patient cohort (spearman rank coefficient: 0.31, p<0.001, figure 3).

### High Plasma Levels of MIF are Associated with the Presence of Plaque Ruptures in ACS Patients

**Figure 4:**
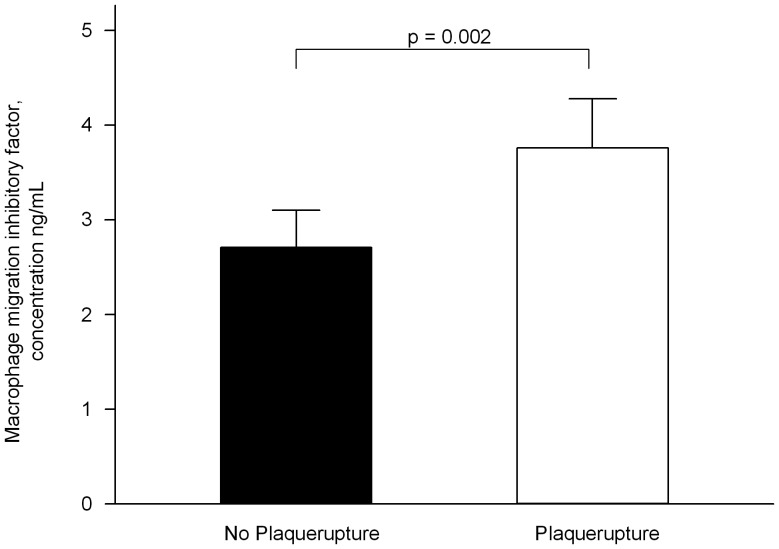
Patients with ACS due to plaque rupture showed higher plasma levels of MIF than patients with flow limiting stenotic lesions in coronary angiography.

Patients with ACS were also analyzed regarding the presence of plaque rupture as verified by coronary angiography. Remarkably, we found that patients with an angiographically verified “culprit” lesion due to vulnerable plaques showed significantly higher plasma levels of MIF compared to patients with ACS caused by flow limiting stenotic lesions (p = 0.002, figure 4). In ROC analysis, we identified a cut off-value of 1.22 ng/mL with a sensitivity of 82% and a specifity of 56% and of 1.7 ng/mL with a sensitivity of 67% and a specifitiy of 62% for the diagnosis of ACS compared to patients with stable CAD and healthy controls. For the diagnosis of plaque rupture in ACS patients we found a high specifity (92%) for MIF levels >5.1 ng/mL.

## Discussion

In the present study we demonstrate that the expression of MIF is significantly enhanced in ACS at an early phase during the event. This effect was independent from other risk factors that were reported to influence the incidence and prognosis of ACS. There is growing evidence that recruitment and migration of inflammatory cells into the vessel wall lead to promotion of atherosclerosis and support the development of unstable atherosclerotic lesions. The different phases of monocyte and macrophage recruitment, adhesion and transmigration are regulated by specifically acting chemokines [11], [12]. MIF has previously been decribed in many other proinflammatory diseases such as arthritis [13], septic shock [14], colitis and hypersensitivity syndrome [15]. MIF is a chemokine-like protein and therefore takes over many chemokine like functions regarding the recruitment and chemotaxis of inflammatory cells and the evolvement of atherosclerotic lesions. Association of MIF with extensive lesion development was previously described in animal models of hypercholesterolemic rabbits [16]. In clinical setting, there are only few reports investigating the association of MIF-expression with markers of plaque instability. The formation of vulnerable plaques in coronary arteries is a critical stage of the atherosclerotic disease with a high risk for acute myocardial infarction. Therefore, it is crucial to understand its mechanisms. Vulnerable plaques are characterized through high inflammatory activity indexed by a high content of inflammatory cells such as monocytes/macrophages and various chemokines that critically contribute to plaque rupture. In a Chinese population, a significant correlation between MIF and activator protein-1 (AP-1) and MMP-9 and -2 concentrations was found [17].

We could show that high plasma levels of MIF are found in ACS patients with “culprit” lesions followed by plaque rupture. These results may suggest that MIF plays an important role in plaque development and stability. Therefore, MIF may serve as a marker of early ACS and of plaque instability.

We also found an association of higher MIF-levels with the expression of established inflammatory markers. Amplification of the inflammatory response is critical for the development of acute coronary events. Additionally, inflammatory response is associated with the outcome after PCI. Previous studies demonstrated an association between the degree of inflammatory response measured by inflammatory markers, platelet hyperactivity and outcome in PCI-patients. We further investigated the role of MIF on cardiac necrosis marker release. Thus, we found a relevant correlation of initial MIF-levels and subsequent maximum amounts of TnI after PCI.

We are aware that the present study is barely hypothesis generating and has its limitations. First of all, it is a cross-sectional study and we did not perform repeated measurements to investigate the stability and time course of MIF plasma concentration. Secondly, we neither investigated markers of plaque instability, nor the prognostic impact of MIF-levels.

In conclusion, the present data underscore the necessity to further characterize the role of MIF for risk marker analysis and its prognostic role in patients with symptomatic CAD undergoing PCI. We demonstrate that there is a highly significant and independent association of increased plasma amounts of MIF in ACS. Additionally, we show that MIF expression correlates with inflammatory response and the extent of cardiac necrosis markers. This finding is of utmost clinical importance as a linkage of inflammatory response, platelet function and adverse cardiovascular outcome including stent thrombosis has been described previously [18]. Thus, multimodal strategies are supported to addresss the question, if anti-inflammatory and plaque-stabilizing approaches can lead to improved prognosis in patients with symptomatic CAD undergoing PCI [19]. Hence, identification of MIF expression can support risk-stratification in multimarker approaches for ACS. Effects of MIF-modulation on cardiovascular outcome should be investigated in the future. Thus, specific molecules targeting MIF have been recently described [20] and might move into the focus of CAD treatment in the future.
